# Notes and News

**Published:** 1901-09-28

**Authors:** 


### NOTES AND NEWS.

Fifty-three deaths from starvation have been recorded in the metropolis in 1900.

Major-General Sir Ian Hamilton will distribute the prizes to the students of St. Thomas's Hospital Medical School on Wednesday, October 2, at 3 p.m.

The introductory address will be delivered at the opening of the Charing Cross Medical School on October 2 by Mr. J. Taylor, F.R.C.S., Professor of Gynæcology at the University of Birmingham.

Professor Burdon-Sanderson strongly endorses Sir John Simon's appeal for sanatoria for consumptive working men, free of cost, that the bread-earning power of the worker may be as speedily regained as possible.

Sympathy with the Boer women and children in the refugee camps does not seem to abate. Two English representatives of the Society of Friends have visited the camp at Barberton. They suggest that English nurses should replace the Dutch nurses in the hospital.

The winter session at St. Mary's Hospital will open on October 1st. The introductory address will be delivered by Dr. William Hill, Aural Surgeon to the Hospital, at three o'clock. The annual dinner of past and present students of the medical school will be held on October 3rd, Colonel A. B. R. Meyers presiding.

The London School Board is now seriously reconsidering its attitude towards the smallpox outbreak. The operations of the health officers have been seriously impeded for lack of the co-operation of the authorities of the Board Schools. In the case of other schools in the infected districts, facilities have been afforded for the examination of the children attending them.

The Produce Commissioner for the New Zealand Government has published particulars as to the careful inspection of the meat which is exported from New Zealand. Indeed no meat can be exported thence unless a certificate is issued by the veterinary officer that at the time of shipment the meat is in good condition and free from disease. It is very satisfactory to know this, as, amongst the lower classes especially, frozen New Zealand meat has an increasingly large sale.

The name of the County and City of Cork Hospital for Women and Children has been changed to The Victoria Hospital for the Diseases of Women and Children.

October 13th is Professor Virchow's eightieth birthday. The Berlin Municipality are going to celebrate the event by devoting £5,000 to the objects of the Virchow Institute. A banquet will also be held on the day.

A Medical man is urging the use of the motor car for consumptives. He states that he has observed a marked benefit from riding on a motor, and recommends that the motor car should be added to the open-air treatment in sanatoria.

Lord Balfour of Burleigh laid the memorial stone of a new general hospital at Stobhill which is being erected by the Glasgow Parish Council. It is to contain 1,500 patients and a nursing staff of 150. The cost is estimated at £200,000.

Scarlet fever is unusually prevalent in Birmingham, and it is found necessary to make arrangements to secure further accommodation. This will probably be arranged by the erection of a temporary pavilion at Yardley Road, and by utilising existing buildings at Lodge Road. By this means 140 extra beds will be available.

The Victoria Hospital at Frome has been opened recently. It contains two wards for four beds, one for three, one for two beds, and one for one bed, situated on two floors, opening into a corridor. Bathrooms adjoin the wards and sanatory annexes with cross ventilated lobbies. Operating room, doctors' room, the usual offices, and a Nurses' Home complete the institution which has been built at a cost of £5,000. It was commenced as a Jubilee commemoration, and is dedicated to Queen Victoria's memory.

A Reference Library and Charities Bureau will be opened on October 1st at The Hospital building, 28 and 29 Southampton Street, London, W.C. It will be open for the use of all interested in hospitals, general charities, and kindred institutions. The want of such a centre of reference has long been felt, and it is hoped that the undertaking will prove of great usefulness to all those who are connected with, or interested in hospitals, the general charities, and kindred institutions. In our next week's issue we shall give an account of the aims and objects of the library and the bureau, and full information regarding the rules and fees for membership.

The trustees of the Frank James Memorial Home for Seamen have offered the home to be bestowed by the Prince of Wales's Hospital Fund, upon any London hospital needing such an institution. The trustees further add to the usefulness of the gift, by an offer to endow it with £10,000. The most useful adjunct to hospital treatment is the Convalescent Home, and we are glad to see that the careful administration of the Prince of Wales's Fund is securing constantly more and more of the recognition which it merits. A neutral body, representing the experience which the Prince's Fund now does, is a most valuable almoner for the public who have not the same means of ascertaining the wants and merits of medical charities.

